# Investigation of Electrical Properties of BiFeO_3_/LDPE Nanocomposite Dielectrics with Magnetization Treatments

**DOI:** 10.3390/polym13162622

**Published:** 2021-08-06

**Authors:** Wei Song, Yu Sun, Tian-Jiao Yu, Yu-Zhang Fan, Zhi Sun, Bai Han

**Affiliations:** 1 Key Laboratory of Engineering Dielectrics and Its Application, Ministry of Education, Heilongjiang Provincial Key Laboratory of Dielectric Engineering, School of Electrical and Electronic Engineering, Harbin University of Science and Technology, Harbin 150080, China; 2020310235@stu.hrbust.edu.cn (T.-J.Y.); sunzhimems@163.com (Z.S.); bhan@hrbust.edu.cn (B.H.); 2 Shandong Electric Power Equipment Co., Ltd., Licheng District, Jinan 250100, China; yuzhang199672@163.com

**Keywords:** nanodielectrics, BiFeO_3_/LDPE, magnetization, electrical properties

## Abstract

**** The purpose of this paper is to study the effect of nano-bismuth ferrite (BiFeO_3_) on the electrical properties of low-density polyethylene (LDPE) under magnetic-field treatment at different temperatures. BiFeO_3_/LDPE nanocomposites with 2% mass fraction were prepared by the melt-blending method, and their electrical properties were studied. The results showed that compared with LDPE alone, nanocomposites increased the crystal concentration of LDPE and the spherulites of LDPE. Filamentous flake aggregates could be observed. The spherulite change was more obvious under high-temperature magnetization. An agglomerate phenomenon appeared in the composite, and the particle distribution was clear. Under high-temperature magnetization, BiFeO_3_ particles were increased and showed a certain order, but the change for room-temperature magnetization was not obvious. The addition of BiFeO_3_ increased the crystallinity of LDPE. Although the crystallinity decreased after magnetization, it was higher than that of LDPE. An AC test showed that the breakdown strength of the composite was higher than that of LDPE. The breakdown strength increased after magnetization. The increase of breakdown strength at high temperature was less, but the breakdown field strength of the composite was higher than that of LDPE. Compared with LDPE, the conductive current of the composite was lower. So, adding BiFeO_3_ could improve the dielectric properties of LDPE. The current of the composite decayed faster with time. The current decayed slowly after magnetization.

## 1. Introduction

In recent years, high-voltage technology has experienced continuous development, and a modern power-grid system has been formed. The reliability and cost-effectiveness of power transmission have been guaranteed. In the field of power transportation across the sea and inland, it needs to be realized with cables made of polymer composite materials [1]. However, in densely populated areas, high-level voltage is needed to deliver electricity. Therefore, cable insulation materials with better electrical performance are needed. Low-density polyethylene (LDPE) is widely used as insulation material, and has excellent insulation properties and good dielectric properties. It has been used in insulated cables in recent decades [2,3]. As LDPE is an insulating material with poor space-charge characteristics, it will show space-charge accumulation characteristics. These characteristics result in a large number of injections of homopolar charges or obvious accumulation of heteropolar charges [4]. Pure polyethylene material cannot meet the requirements of high-voltage transmission. The use of nanomaterials can have a great impact on the insulation performance of LDPE. When non-conductive fillers are used, the DC conductivity of nanocomposite dielectrics is often low. For example, the filling of MgO and Al_2_O_3_ can significantly reduce the DC conductivity of LDPE [5,6]. At the same time, the deep traps introduced by nanoparticles will block the injection of homopolar space charges and the migration of heteropolar space charges. There are three main differences between nanocomposites and traditional composites: (1) nanocomposites contain a small amount of fillers—the mass fraction of fillers usually is less than 10 wt%, while composites contain more than 50 wt%; (2) the size of fillers is in the nanometer range; and (3) nanocomposites have a large specific surface area [7]. Therefore, nanocomposites have unique advantages such as uniform structure, no fiber fracturing, and good processability. In most of the studies on the dielectric properties of nanomaterials, the in situ method is usually used to prepare samples [8], which can improve the dispersion of nanoparticles and the properties of materials. At present, MMT, SiO_2_, ZnO, MgO, and BaTiO_3_ are the main inorganic nanoparticles used in LDPE insulating materials. At the same time, multiferroic materials have also been widely considered, including bismuth ferrite (BiFeO_3_), which is a typical perovskite (ABO_3_) multiferroic material at room temperature. Pure BiFeO_3_ has a rhombic lattice and a cycloidal spin arrangement that are stable in a wide range of magnetic fields [9]. Because BiFeO_3_ can control spontaneous magnetization through an electric field and spontaneous polarization through a magnetic field, it shows a certain magnetoelectric coupling effect. At the same time, BiFeO_3_ has spontaneous polarization up to 100 μC/cm^2^ [10]. So, it has become one of the most promising candidate materials [11,12,13,14,15,16]. Magnetic-field treatment can make BiFeO_3_ exhibit a helical spin structure with a period of 62 nm [17]. For this structure, BiFeO_3_ exhibits weak magnetization. The trap generated by adding nano-bismuth ferrite particles will capture the charge and limit its movement. This trap can prevent the accumulation of the charge and evenly distribute it. Thus, the excitation of hot electrons is hindered. It is conducive to improving the insulation and dielectric properties of LDPE [18]. For example, due to the magnetic-field-induced arrangement of multi- and single-walled carbon nanotubes in a polycarbonate matrix, the I-V properties of carbon nanotubes and polycarbonate nanocomposites showed that conductivity was improved [19]. This shows that the magnetic-field treatment has a great influence on magnetic composites.

In this paper, 2 wt% nano-BiFeO_3_/LDPE composites were prepared by the melt-blending method. The composite contained LDPE as the matrix and nano-BiFeO_3_ as the filler. The composite was then magnetized. The surface of the BiFeO_3_ nanoparticles was observed using a scanning electron microscope. The crystallinity of the composite was measured by thermogravimetry differential scanning calorimetry (DSC). The breakdown field strength was measured using a breakdown device. The conductance current and I-t characteristics were measured with an EST122 picoammeter and an aluminum three-electrode system. The space charge was measured with the electroacoustic pulse method. The difference between BiFeO_3_/LDPE composites and LDPE was studied by testing the properties of the materials.

## 2. Experiments

### 2.1. Material Synthesis

In the experiment, the ferric nitrate nonahydrate (Fe(NO_3_)_3_·9H_2_O), bismuth(III) nitrate pentahydrate (Bi(NO_3_)_3_·5H_2_O), citric acid monohydrate (C_6_H_8_O_7_·H_2_O), nitric acid, distilled water, ammonia, and absolute ethanol were all provided by Tianjin Kemiou Chemical Reagent Co., Ltd. (Tianjin, China). The LDPE was purchased from Sinopec Beijing Yanshan company(Yanshan, China). The preparation of BiFeO_3_ nanoparticles is described in reference [3].

After weighing 98 g of dried LDPE and 2 g of nano-BiFeO_3_, the BiFeO_3_ was mixed with an appropriate amount of anhydrous ethanol. The BiFeO_3_ was evenly dispersed in ethanol with an ultrasonic instrument (FS-300, Shanghai Shengxi Ultrasonic Instrument Co., Ltd. Shanghai, China). The LDPE was melted at 120 °C using a torque rheometer (Hapro Electric Technology Co., Ltd. Harbin City, Heilongjiang Province, China.) for 10 min. Then, the BiFeO_3_ and LDPE were mixed at the same temperature for 10 min. Finally, the BiFeO_3_/LDPE composite was obtained. The BiFeO_3_/LDPE composite was placed on the plate vulcanizer (XLB25-D, Zhejiang Shuangli Group Huzhou Xingli Rubber Machinery Manufacturing Co., Ltd. Huzhou City, Zhejiang Province, China.). At 120 °C, the sample needed to be pressurized at 5 MPa for 5 min, 10 MPa for 10 min, and 15 MPa for 15 min. Test samples with a 100–300 μm thickness were obtained.

The flake BiFeO_3_/LDPE composite samples pressed by flat vulcanizer and die (processed with non-ferromagnetic materials) were put into the stable magnetic field generator (SBV220, Changchun Yingpu Magnetoelectric Technology Development Co., Ltd. Changchun City, Jilin Province, China.). The diameter of the magnetic head was 100 mm. The temperature of the magnetic head was between room temperature and 200 °C. The magnetic field direction was perpendicular to the desktop. When the samples were magnetized at room temperature, the magnetic field intensity was adjusted to 1.5 T, and the samples were magnetized for 15 min. When samples were magnetized at high temperature, the magnetization temperature was 100 °C, the magnetic field was adjusted to 1.5 T, and the samples were magnetized for 15 min. Then, the heating was stopped to let the sample cool to 25 °C while still under a magnetic field strength of 1.5 T. The samples were then removed from the magnetic field generator. The samples magnetized at room temperature were recorded as R-M, and the samples magnetized at high temperature were recorded as H-M.

### 2.2. Characterization and Testing Methods

Material characterization instrument model and test conditions: An atomic force microscope (AFM) was used to observe the smooth specimen with a diameter of 1 cm and thickness of 300 μm in tapping mode. An S-4800 scanning electron microscope was used. The test conditions were: cold field emission electron source, acceleration voltage of 0.5–20 kV, and amplification factor of 5–45 k. At the same time, it was necessary to freeze the BiFeO_3_/LDPE composite flakes in liquid nitrogen. Then, the samples were broken and pasted on the sample table to observe the fracture morphology. The samples of BiFeO_3_/LDPE composite flakes were tested by using the German NEZSCHSTA449c thermogravimetry differential scanning calorimetry analyzer. The weight of the samples ranged from 5 mg to 10 mg. The temperature range of the test was 50–150 °C. The heating rate was 5 °C/min. The polyethylene nanocomposites were then characterized.

Material performance test and experimental conditions: In the breakdown experiment, the thickness of the sample was 100 μm. The voltage was raised at a rate of 1 kV/s until the breakdown of the material occurred, then the data were recorded. The upper and lower diameters of the electrodes used in the experiment were 25 mm and 50 mm, respectively. The electrodes and samples for the breakdown test were required to be immersed in dimethyl silicone oil. Each type of LDPE and composite material was tested 10 times.

The conductance current was measured with an EST122 picoammeter and an aluminum three-electrode system. The thickness of the samples selected during the experiment was approximately 100 μm. In order to control the ambient temperature of the experiment, the drying process was carried out during the test and analysis. After drying for 1 h, the increasing-voltage experiment was carried out. The electric field strengths used were 5 kV/mm, 7.5 kV/mm, 10 kV/mm, 15 kV/mm, 20 kV/mm, 25 kV/mm, 30 kV/mm, and 35 kV/mm. We then determined the current value and obtained the conductance current.

I-t characteristics were measured by using the EST122 picoammeter, the three-electrode system, and a Dongwen high-voltage DC power supply. The minimum range of the picoammeter was 10^−14^ A. The maximum range of high-voltage DC power supply was 20 kV. The thickness range of the test samples was 100 μm. The upper and lower surfaces were plated with three electrodes. Before the test, the samples were short-circuited in an 80 °C oven for 24 h, then they were placed into the three-electrode test system. The samples required the short-circuit treatment at room temperature for 30 min until the current decayed below 0.1 pA, then the test began. Constant DC electric fields of 10 kV/mm and 30 kV/mm were applied in the measurement. The polarization current value was collected by a data-acquisition card. The sampling frequency was 10 Hz.

The space charge was measured with the electroacoustic pulse method. A sample with a thickness of 300 μm was subjected to a DC electric field of 40 kV/mm at room temperature by means of gradient voltage. The space-charge distribution was measured after 10 min of treatment.

## 3. Results and Discussion

### 3.1. Molecular Structure Characterization

**Figure 1:**
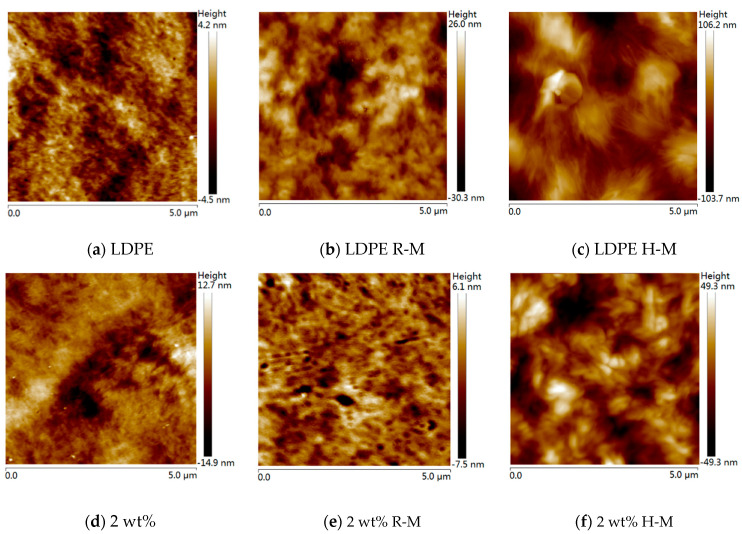
AFM images of the LDPE (**a**–**c**) and 2 wt% composites (**d**–**f**) with height maps.

The AFM test image with a length and width of 5 μm was obtained through the test. Figure 1 shows that the spacing between the lamellar crystals of LDPE obviously increased after magnetization. The enlargement of spherulites can also be clearly observed. The results showed that 2 wt% nano-BiFeO_3_ particles played the role of nucleating agent after filling into the LDPE, which improved the crystal concentration of the LDPE matrix. The spherulites of the composite became much larger. The spherulite of the composite was larger than that of the LDPE, and the filamentous flake aggregates could be observed more clearly. The spherulites became obviously larger under high-temperature magnetization. Among them, the spherulites’ size ranges in unmagnetized LDPE, LDPE magnetized at room temperature, and LDPE magnetized at high temperature were 0.2–0.6 μm, 0.5–0.7 μm, and 1–1.25 μm, respectively; and the spherulites’ sizes in the composite material were 0.3–0.7 μm, 0.5–1 μm, and 0.7–2 μm, respectively. It can be seen in Figure 1 that the size of the spherulites increased, since the number of spherulites shown in the picture became less. After high-temperature magnetization, the LDPE and composite wafers became thicker, and the distance between the composite wafers became larger.

**Figure 2:**
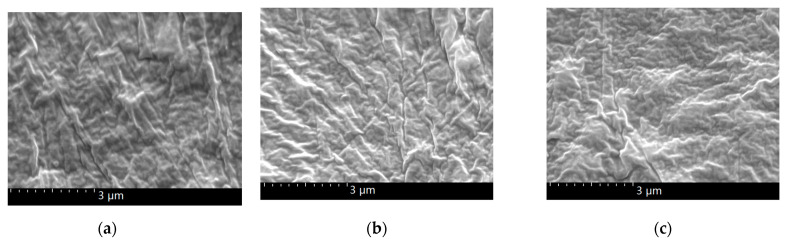
SEM of LDPE sections: (**a**) LDPE; (**b**) LDPE R-M; (**c**) LDPE H-M.

**Figure 3:**
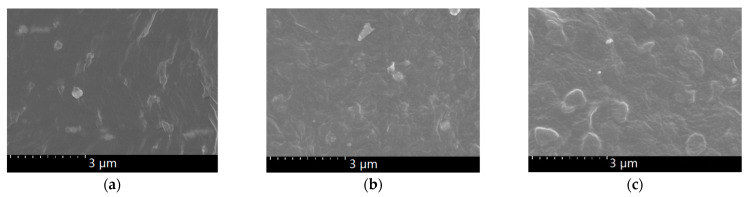
SEM of composite sections: (**a**) 2 wt%; (**b**) 2 wt% R-M; (**c**) 2 wt% H-M.

Figure 2 shows the contrast SEM of the LDPE before and after magnetization. As can be seen in the figure, magnetic-field treatment had little effect on the morphology of the LDPE. The reason is that LDPE has a homogeneous structure, so there was no interface problem. Figure 3 shows that the BiFeO_3_ particles agglomerated in the LDPE matrix, since the particles are clearly visible. After high-temperature magnetization (Figure 3c), there were BiFeO_3_ nanoparticles following a certain direction on the section, and the number of nanoparticles was more than can be seen in Figure 3a,b. The reason is that when they were magnetized at high temperature, there was enough space for the free arrangement of the nanoparticles and LDPE matrix. Under the action of the magnetic field, there were magnetic dipoles in the magnetic particles, which interacted with each other to form a multipolar magnetic moment. The magnetic force between particles was anisotropic, which made their magnetic moment arrange end to end, so that the particles were close to each other [20]. However, this phenomenon cannot be seen in Figure 3b (room-temperature magnetization). The reason was that it was difficult for the macromolecules of the LDPE matrix to move, and the nanoparticles could not be rearranged when magnetized at room temperature. Therefore, the SEM images of samples magnetized at room temperature are similar to those of unmagnetized samples.

**Figure 4:**
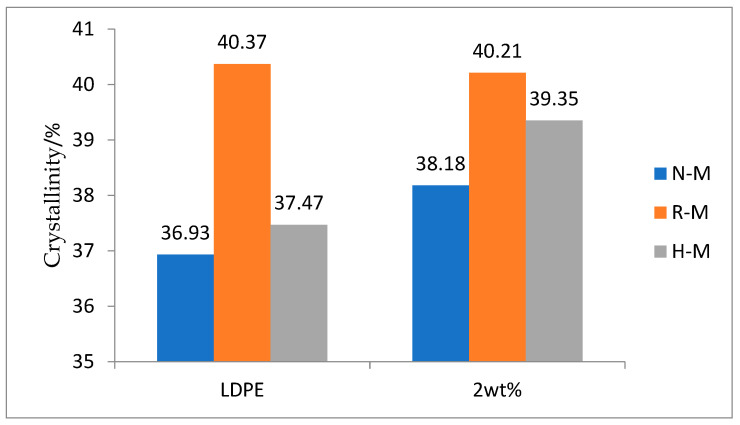
Comparison of the crystallinity of the LDPE and the 2 wt% composites.

The left side of Figure 4 shows a DSC histogram of pure LDPE, room-temperature magnetization, and high-temperature magnetization. When considered with the data in Table 1 (N-M: unmagnetized), we found that the crystallinity of the LDPE increased after magnetization at room temperature or high temperature. The reason was that the magnetic field changed the intermolecular and intramolecular forces of the LDPE, and the orientation of the macromolecules was easily changed [21]. The orientation was enhanced, and anisotropy was induced along the direction of the magnetic field. The alignment of the LDPE chains or bundles became more regular, which led to the increase in crystallinity of the samples after magnetization. In the case of high-temperature magnetization, the ability of the molecular motion was enhanced and it was easier to induce directional alignment under the action of the magnetic field. Therefore, the crystallinity of the LDPE samples magnetized at high temperature had a greater increase.

The right side of Figure 4 shows a DSC histogram of the nanocomposite with 2 wt %. Compared with Table 1, it can be seen that for the 2 wt% nanocomposites, the crystallinity of the composites magnetized at room temperature was higher than that of the unmagnetized samples. At the same time, the increase in crystallinity of the samples magnetized at a high temperature was less than that of the samples magnetized at room temperature. The reason was that LDPE is a soft material, and macromolecular chains easily respond to changes in the surrounding environment and external field. BiFeO_3_ is an antiferromagnetic material. The magnetization process of BiFeO_3_ in an external magnetic field is mainly a turning process of a magnetic dipole moment in a magnetic domain. The magnetic dipole moment is easily oriented along the direction of the external magnetic field, which plays a traction role in the surrounding LDPE and promotes the nucleation. At a high temperature, the viscosity of LDPE matrix increased, the spherulites of LDPE were elongated along the direction of magnetic field, the nucleation was inhibited, and the spherulites became larger. As a result, the increase in crystallinity was low after high-temperature magnetization. The AFM images also show that the spherulites became larger after magnetization, so we concluded that the addition of nano-BiFeO_3_ particles and magnetization treatment increased the crystallinity of LDPE.

### 3.2. Breakdown Field Strength

**Figure 5:**
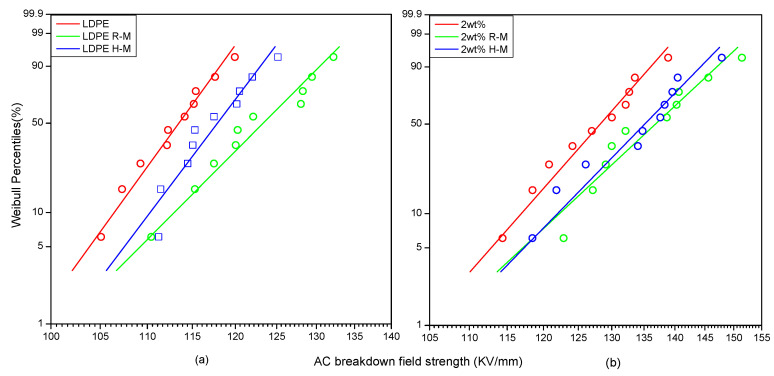
Weibull distribution of AC breakdown between the LDPE and composites under different magnetization conditions. (**a**) is the Weibull distribution of LDPE. (**b**) is the Weibull distribution of BiFeO_3_/LDPE composite. Curves LDPE and 2 wt% indicate no magnetization treatment. R-M is room temperature magnetization. H-M is high temperature magnetization.

The change of breakdown field strength between the 2 wt% BiFeO_3_/LDPE composite and pure LDPE was analyzed. Figure 5a shows the Weibull distribution of the AC breakdown of the LDPE under different magnetization conditions. It can be observed that the AC breakdown strength of the LDPE increased significantly after being treated with a stable magnetic field at room temperature. After high-temperature magnetization, the AC breakdown field strength decreased slightly, but was still higher than that of the LDPE. It can be seen in Figure 5b that the breakdown strength of samples magnetized at room temperature was higher than that of the unmagnetized samples, and that of the samples magnetized at high temperature was lower than that of the samples magnetized at room temperature. However, the breakdown strength was higher than that of the LDPE. The breakdown field strength of the composite was 13.5% higher than that of the LDPE. The breakdown field strength of the two materials increased by 8.1% under room-temperature magnetization. The breakdown field strength of the two materials increased by 4.7% under high-temperature magnetization. The reason was that the arrangement of the doped BiFeO_3_ particles was more orderly after magnetization treatment, which made the electrons more easily trapped by the deep traps in the composite. Hence, the breakdown strength of the composite was improved.

### 3.3. Conductance Current Test

**Figure 6:**
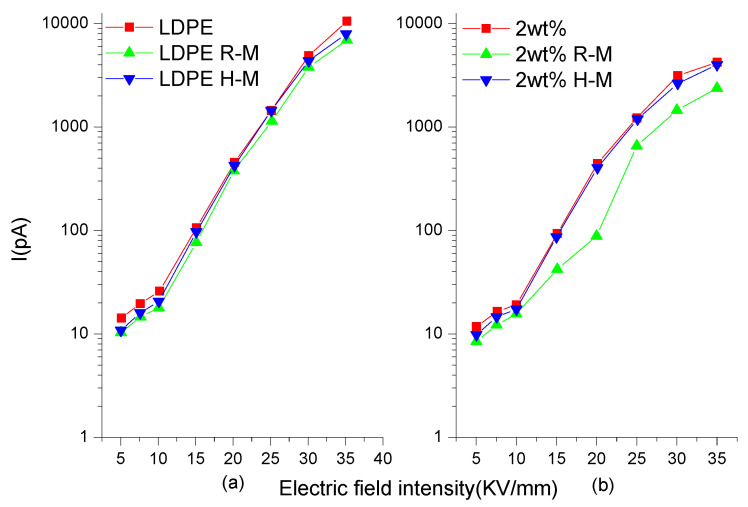
Conductivity current characteristic curves of LDPE (**a**) and composites (**b**)at different magnetization temperatures. (**a**) is the conductivity current characteristic curve of LDPE. (**b**) is the conductivity current characteristic curve of BiFeO_3_/LDPE composite. Curves LDPE and 2 wt% indicate no magnetization treatment. R-M is room temperature magnetization. H-M is high temperature magnetization.

Figure 6a,b show the conductivity current characteristic curves of the LDPE and composites at different magnetization temperatures. It can be seen in Figure 6a that after room-temperature magnetization and high-temperature magnetization treatment, the change in conductivity current for the LDPE was not obvious, and the conductivity current only decreased slightly compared with that of the LDPE without magnetization treatment. This was because LDPE is a non-polar material, and its main conductivity form is ionic. Magnetization treatment can improve the crystallinity of LDPE, increase the area of crystalline region, and decrease the size of amorphous region. Carriers mainly transport in the amorphous region, which is not conducive to ion migration, and leads to the decrease of ionic conductivity. It can be seen in Figure 6b that the conductance current decreased after magnetization. This was because high-temperature magnetization led to recrystallization in the composite, which led to a change in internal structure and an enrichment effect. This led to the deterioration of the conductivity of the composite.

### 3.4. I-t Characteristics

The current generated by applying DC voltage to dielectric materials is divided into three parts:

(1) $$I=I_{sp}+I_{a}+I_{d}$$

where $I_{sp}$ is the geometric capacitor-charging current and the fast charging current caused by instantaneous polarization, $I_{a}$ is the absorption current caused by slow polarization and space charge formation, and $I_{d}$ is the steady-state conductance current.

**Figure 7:**
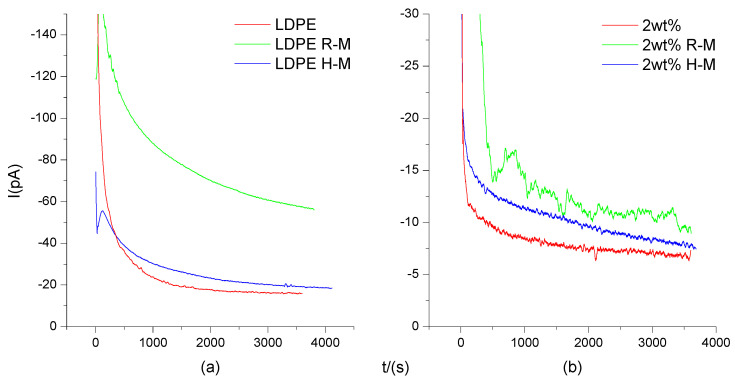
I-t characteristic curve of LDPE (**a**) and composites (**b**) under a 10 kV/mm electric field strength and different magnetization temperatures. (**a**) is the I-t characteristic curve of LDPE. (**b**) is the I-t characteristic curve of BiFeO_3_/LDPE composite. Curves LDPE and 2wt% indicate no magnetization treatment. R-M is room temperature magnetization. H-M is high temperature magnetization.

As can be seen in Figure 7, under an electric field intensity of 10 kV/mm, the current of LDPE and composites decreased with the increase of time. The attenuation of the LDPE current was slower than that of the composites’ current. The attenuation of the magnetized samples’ current was slower than that of the unmagnetized samples current. When the electric field intensity was less than 10 kV/mm, the injection of space charge was very small. Therefore, the current was mainly determined by the fast charging current, the slow polarization current, and the steady-state conductance current. The polarization current included interface polarization, BiFeO_3_ magnetic domain switching polarization and, hot ion polarization. The polarization current decayed with time.

**Figure 8:**
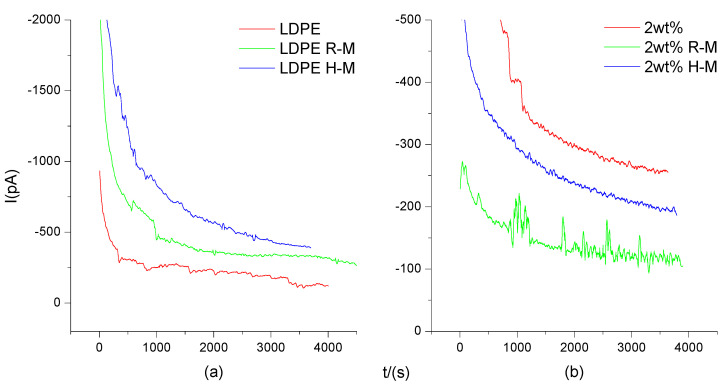
I-t characteristic curve of LDPE (**a**) and composites (**b**) under a 30 kV/mm electric field strength and different magnetization temperatures. (**a**) is the I-t characteristic curve of LDPE. (**b**) is the I-t characteristic curve of BiFeO_3_/LDPE composite. Curves LDPE and 2 wt% indicate no magnetization treatment. R-M is room temperature magnetization. H-M is high temperature magnetization.

As can be seen in Figure 8, under a high field of 30 KV/mm, the current values for the LDPE and composites increased, and the current of the composites changed little with time. The conductivity of the composites decreased after magnetization. The reason was that more space charges were generated in the composites under high electric field, and the impurities in the composites were ionized. The longer the time, the more the impurities were ionized. However, magnetization treatment can lead to the enrichment effect of nanoparticles, reducing the density of the conductive network and reducing the conductive current.

### 3.5. PEA Space-Charge Test

**Figure 9:**
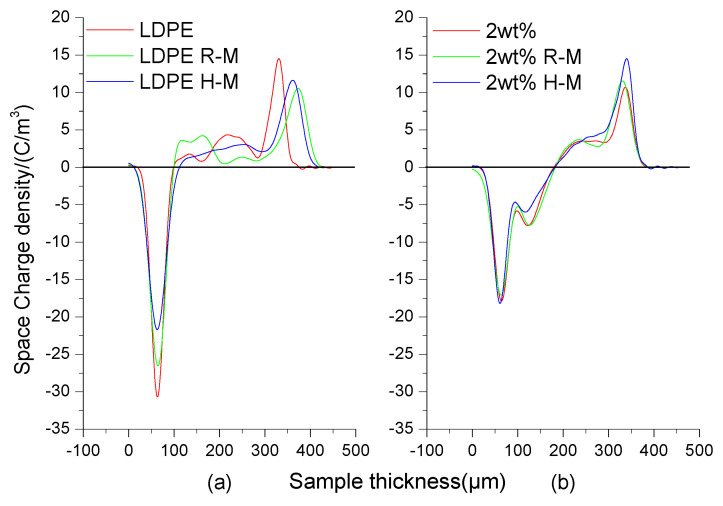
Test curve at 40 KV showing the space charge of LDPE (**a**) and composites (**b**) at different magnetization temperatures. (**a**) is the space charge curve of LDPE. (**b**) is the space charge region curve of the BiFeO_3_/LDPE composite. Curves LDPE and 2 wt% indicate no magnetization treatment. R-M is room temperature magnetization. H-M is high temperature magnetization.

As can be seen in Figure 9, under an electric field of 40 kV/mm, the space-charge accumulation of the LDPE was obvious, the space-charge accumulation of the unmagnetized LDPE was the most obvious, and the space-charge accumulation of the room-temperature-magnetized and high-temperature-magnetized LDPE was less. The reason was that the LDPE was more susceptible to the effect of the external magnetic field producing induced dipoles in the molten state. The molecular chain arrangement was more orderly. The crystallinity increased, and crystallization tends to be directional. The space-charge accumulation of the BiFeO_3_/LDPE nanocomposites was less. This was because the addition of nano-BiFeO_3_ increased the interface region of the composites, and provided more dense trap energy levels and numbers. At the same time, the restricted current carrier induced a homopolar charge aggregation in the local region of the polymer. Since this phenomenon forms a reverse electric field, which partially counteracts the applied electric field, it improves the electric field strength of charge injected into the material from the electrode. So, magnetization had little effect on the space charge.

## 4. Conclusions

Nano-BiFeO_3_/LDPE composites were prepared by melt blending. LDPE and composites were treated with a magnetic field at different temperatures. Their dielectric properties were improved. After magnetization treatment, the spherulites of the nano-BiFeO_3_/LDPE composites became larger, and the crystallinity increased. This increase in crystallinity led to a decrease in the amorphous region, and was not conducive to ion migration. This led to a decrease in conductance current. After magnetization, the nano-BiFeO_3_ particles were arranged regularly, which could induce traps to capture electrons to improve the AC breakdown field strength. The polarization current was produced after magnetization treatment in a low field, which caused the current to decay more slowly with time. In a high field, the current increased due to higher space charges, but it could be reduced by magnetization. Compared with LDPE, the traps introduced by the nano-BiFeO_3_ led to the accumulation of homopolar charges, and formed a reverse electric field. This led to lower space-charge accumulation in the composites under a high field. This improved the obvious disadvantage of LDPE space-charge accumulation. In this paper, a new preparation method was adopted to change the crystallinity and crystalline morphology of the material by subjecting the material to a magnetic field at different temperatures, thereby regulating the dielectric properties of the composite material. This study provides a method for the modification of LDPE.

## Figures and Tables

**Table 1:** Crystallinity of LDPE and 2 wt% composites.

Sample Name	Peak (°C)	Delta H (J/g)	Crystallinity (%)
LDPE LDPE R-M LDPE H-M 2 wt% 2 wt% R-M 2 wt% H-M	107.55 106.61 107.57 107.20 108.31 107.52	−108.44 −118.52 −110.01 −110.34 −118.07 −115.54	36.93 40.37 37.47 38.18 40.21 39.35
